# Chemical carving lithography with scanning catalytic probes

**DOI:** 10.1038/s41598-020-70407-1

**Published:** 2020-08-07

**Authors:** Bugeun Ki, Kyunghwan Kim, Keorock Choi, Jungwoo Oh

**Affiliations:** 1 School of Integrated Technology, Yonsei University, Inchon, 21983 Republic of Korea; 2 Yonsei Institute of Convergence Technology, Inchon, 21983 Republic of Korea

**Keywords:** Engineering, Nanoscience and technology

## Abstract

This study introduces a new chemical carving technique as an alternative to existing lithography and etching techniques. Chemical carving incorporates the concept of scanning probe lithography and metal-assisted chemical etching (MaCE). A catalyst-coated probe mechanically scans a Si substrate in a solution, and the Si is chemically etched into the shape of the probes, forming pre-defined 3D patterns. A metal catalyst is used to oxidize the Si, and the silicon oxide formed is etched in the solution; this local MaCE reaction takes place continuously on the Si substrate in the scanning direction of probes. Polymer resist patterning for subsequent etching is not required; instead, scanning probes pattern the oxidation mask directly and chemical etching of Si occurs concurrently. A prototype that drives the probe with an actuator was used to analyze various aspects of the etching profiles based on the scanning speeds and sizes of the probe used. This technique suggests the possibility of forming arbitrary structures because the carving trajectory is formed according to the scan direction of the probes.

## Introduction

In order to maximize a semiconductor’s performance per cost, the density of chips on each wafer has been increased exponentially. 3D semiconductor structures are considered as indispensable solution in the last decade and made by fabricating photoresist patterns via lithography followed by dry etching. Conventional photography and nanoimprint lithography require masks and molds with pre-defined patterns. Electron-beam lithography can directly draw detailed patterns at a desired nano-scale, which is used for special applications^1–3^. However, it is difficult to meet both productivity and resolution requirements using these lithography technologies owing to the use of a single beam or limitations in mask precision. They also involve complex process steps and high-cost optical designs using photoresists, which react to light or an electron beam, and are subject to subsequent contamination. There is an increasing demand for patterning technology for fabricating nanoscale 3D structures, which in turn increases the complexity and cost of the processing equipment involved.

Studies have been performed on alternative technologies that can be used to quickly form patterns while maintaining a high resolution. The scanning probe lithography (SPL) process adds or removes material to/from the substrate while directly drawing a pattern with a probe^4,5^. Precise patterns with a resolution less than 10 nm can be created based on the size of the probe tip. Thermal scanning probe lithography (t-SPL) technology uses heat to remove polymer resists on the substrate, and it is the fastest for drawing patterns from the various SPL methods^6–9^. An SPL process that uses a probe array can easily create precise patterns over a large area simultaneously^10–13^. In addition, studies have been conducted on SPL technologies that reduce the number of process steps involved. Oxidation scanning probe lithography (o-SPL) directly oxidizes the semiconductor substrates^14–16^ and the subsequent etching process does not require polymer resist patterns, which simplifies the overall lithography and etching processes. These techniques can be used to quickly draw precise patterns successively, and they have attracted a lot of attention in academia.

A 3D semiconductor structure can be fabricated by forming a mask pattern on a substrate via lithography and then subjecting it to an etching process. Dry etching is advantageous for anisotropic etching in order to fabricate 3D structures with a high aspect ratio. Reactive-ion etching (RIE) is used to create uniform structures with high aspect ratios over large areas. However, the crystalline semiconductor tends to experience structural damage because of high-energy plasma ions^17,18^. This process is more difficult than others due to the different etching characteristics based on the pattern size and shape, and it requires the use of toxic gas facilities and vacuum equipment.

Metal-assisted chemical etching (MaCE) technology is being studied as an alternative to this technology^19–22^. Since MaCE involves only a chemical reaction between the metal catalyst and substrate in the etching solution, it imprints patterns directly on the semiconductor without affecting its crystallinity. It is advantageous because it is an anisotropic etching technique that can produce 3D structures with a large aspect ratio even though only chemical reactions are involved. In addition, it can lower production costs because it does not require expensive and complex gas facilities and vacuum equipment. However, the metal used as the catalyst remains in the semiconductor and must be removed after MaCE, indicating that it is not possible to recycle the precious metals used.

**Figure 1:**
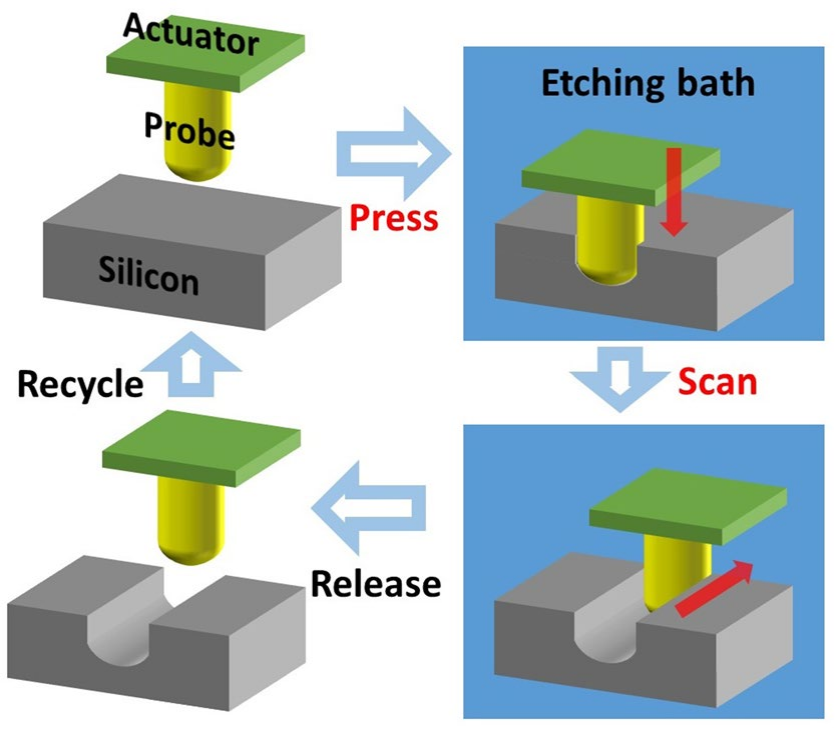
Schematic of chemical carving of crystalline Si substrates, which integrates scanning probe lithography and metal-assisted chemical etching. The metal-catalyst-coated probe scans the Si substrate in the etching solution, and the Si is chemically etched in the shape of the probe to form three-dimensional patterns.

This paper presents chemical carving as a new patterning method in which Si substrates are carved via chemical reactions involving catalytic probes. Chemical carving begins when contact is made between the metal catalyst probe and Si substrate in an etching solution consisting of an oxidant and acid (Fig. 1). As pressure is applied only in the vertical direction, the etching progresses toward the bottom of the substrate^23–28^. Then, when the probe scans sideways, the side of the semiconductor substrate that touches the probe is etched. The metal deposited on the probe catalyzes the reduction of the oxidant in the etching solution. Electronic holes (h^+^) injected into the substrate oxidize the Si, and then, the acid in the solution dissolves the Si oxide. As probe scan moves toward the side, the substrate is etched concurrently and 3D structures are fabricated. After the probe is separated from the Si, it can be recycled for use in the next round.

The chemical carving method proposed in this work is a new patterning technique that combines existing SPL and MaCE processes for fabricating arbitrary patterns in a simplified way. It can be used to produce a 3D semiconductor structure without a mask or polymer resist. Since it uses chemical reactions for carving, it does not damage the crystal surface. The metal catalyst can be reused because it is attached to the probe, unlike in conventional MaCE where it must be removed after one use. In addition, it allows for greyscale lithography, which can be used to adjust the etching depth according to the scan speed. The concept demonstrated with prototype equipment can be further refined through the development of precision equipment and durable probe materials.

## Result and discussion

**Figure 2:**
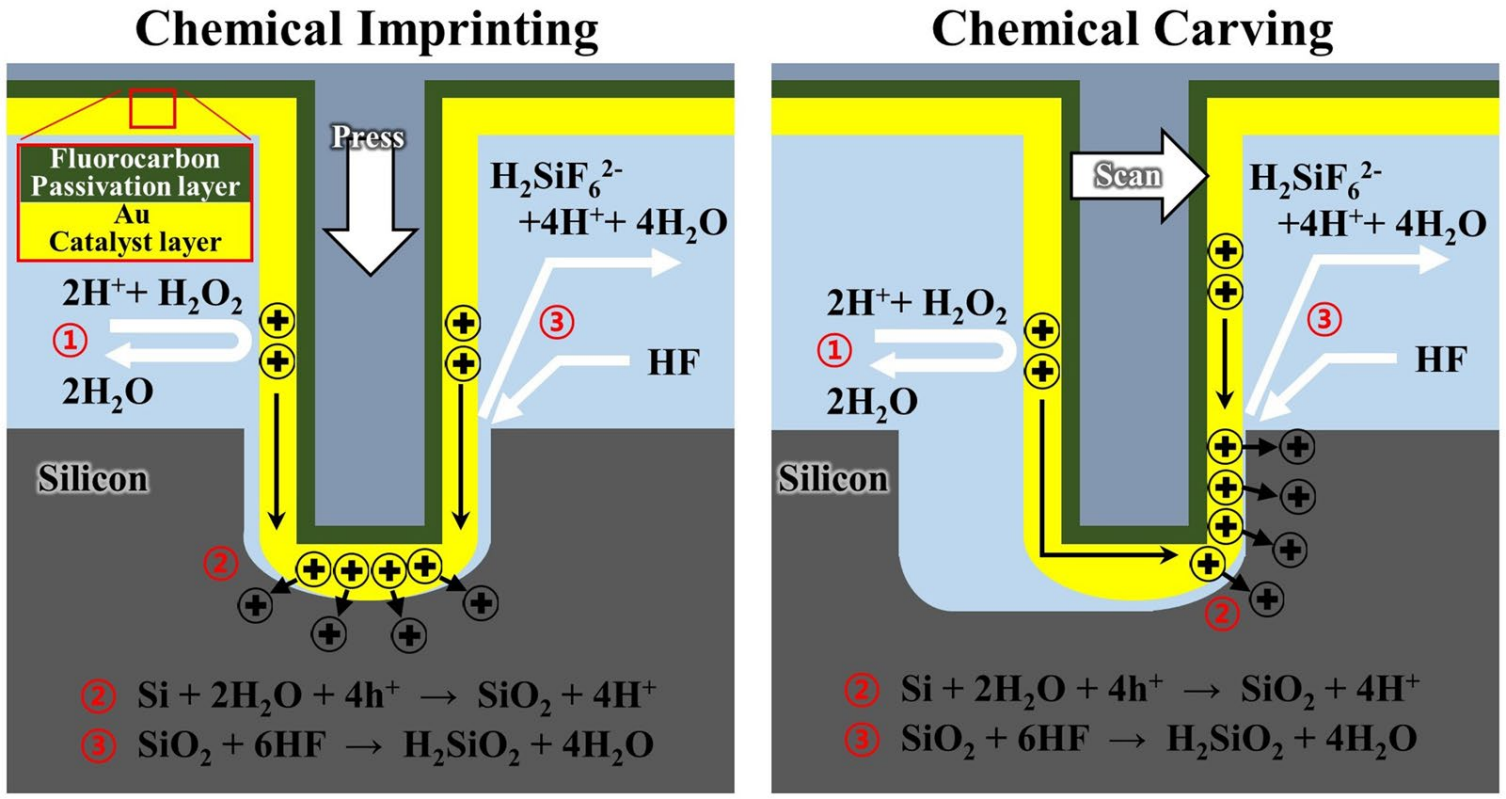
Schematic of chemical imprinting and chemical carving mechanisms. In both cases, etching occurs as follows: (1) the Au layer catalyzes the reduction of H_2_O_2_ to H_2_O in the etch bath. As a result of the reduction, electronic holes (h^+^) are formed in the Au layer. (2) The holes (h^+^) are transferred and injected through the Au layer into the Si substrate. The Si is locally oxidized. (3) The oxidized Si is dissolved by HF in the solution. The Au/Si gap formed after etching is brought into Au/Si contact again due to the actuator’s force in the vertical and horizontal directions.

Figure 2 shows the chemical carving mechanism. The metal-catalyst-coated probe comes into contact with the Si substrate in an etching bath consisting of an oxidant and an acid. As the probe catalyzes the reduction of the oxidant, electronic holes (h^+^) are transferred to the metal on the probe (1). These holes (h^+^) are then injected into the Si and oxidation occurs (2). The oxidized Si is dissolved by the acid in the solution, and a series of reactions continue to occur in the direction of probe scanning (3).

The holes are formed by pressing the probes in the vertical direction during the above-mentioned continuous reaction, which we call chemical imprinting. After the holes are formed, when the actuator scans the probe in the horizontal direction, trench-like etching occurs, which is called chemical carving in this study. A probe embedded in the hole is scannto the probe to cause faster breakdowned in the horizontal direction and a chemical reaction with Si occurs, which results in the continuous etching of the side of the Si to form a trench. The etching occurs in the direction that the metal-catalyst-coated probe repeatedly comes into contact with the side of the semiconductor.

Both chemical imprinting and carving are possible because the metal catalysts are deposited on the bottom as well as the side of the probe. Since the metal catalyst is not attached to the Si as in conventional MaCE, the probe and oxidized Si are separated instantly once chemical etching occurs, which suggests that a stationary probe cannot etch the Si substrate continuously. In the proposed technique, the actuator applies a constant force in the vertical and horizontal directions to bring the gap formed back into Au/Si contact after etching, which results in continuous etching (Fig. S1).

**Figure 3:**
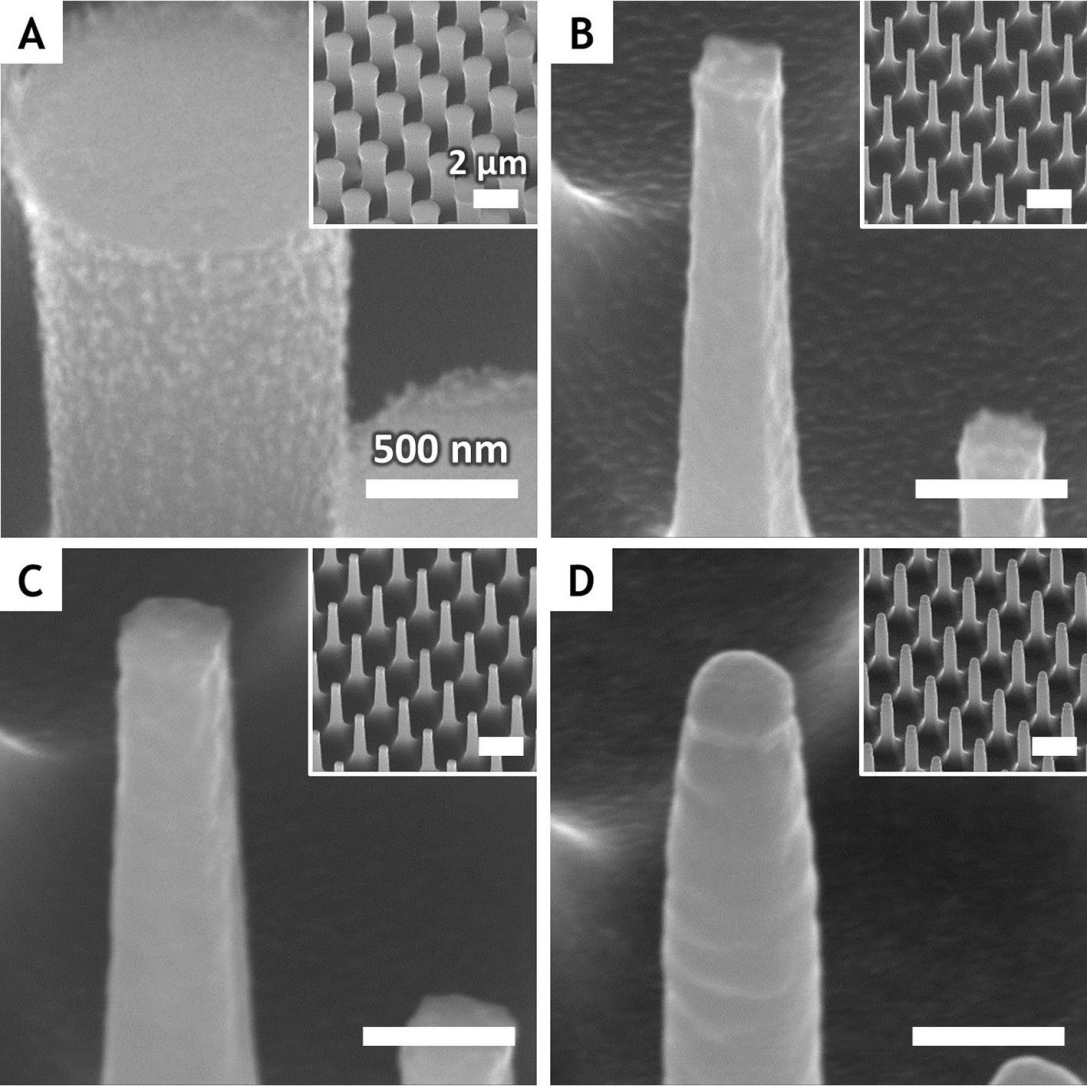
SEM images of probe array fabricated using standard semiconductor processes. (**A**) High-aspect-ratio circular pillars were dry-etched on Si substrates using ICP-RIE and Bosch process. (**B**) The diameter of the circular pillars was reduced to 300 nm using ICP-RIE after resist removal. (**C**) A 50-nm-thick fluorocarbon layer was deposited for electrochemical passivation to prevent the probes from being etched. (**D**) A 100-nm-thick layer of Au catalyst was sputtered on the probes for MaCE.

Figure 3 shows molds with probe arrays fabricated using a standard semiconductor process on a Si substrate. Starting with a photolithographic polymer pattern of a diameter of 1 μm, a pillar-shaped probe was fabricated using ICP-RIE and a metal catalyst were sputtered. The aspect ratio of the probe was increased using the Bosch process and a pillar array with a height of 5 μm was fabricated (Fig. 3A). Under the given conditions, the diameter of the pillar was trimmed to 300 nm after the photoresist was removed to reduce the probe size (Fig. 3B).

If the metal catalyst and Si probe are in direct contact, holes (h^+^) are injected into the Si that oxidize the Si probe. As a result, the probe is etched rather than the target substrate during chemical carving. As a result, the probe is etched rather than the target substrate during chemical carving. To prevent such unwanted etching of the probe itself, an electrochemical protective layer was inserted between the probe and metal catalyst (Fig. 3C). A 50-nm-thick fluorocarbon layer was deposited using C_4_F_8_ gas plasma via ICP-RIE, which has proven to be an effective electrochemical protective layer during chemical imprinting^27,28^.

The probe was attached to a holder fixed to a vertical actuator. The target substrate was placed in an etch bath above a motorized stage so that the probe can scan the target substrate in the etching solution. The etching solution consisted of HF, H_2_O_2_, and deionized (DI) water with a ratio of 10:1:10, and the process was performed at room temperature. It has been reported that MaCE is affected by mass transfer of reactants and products during chemical reactions, which results in more uniform etching results for finer patterns. If the probes are created using e-beam lithography used in existing nano-imprint lithography (NIL) or SPL, more dramatic results such as nanometric structures with higher aspect ratio can be expected in future.

**Figure 4:**
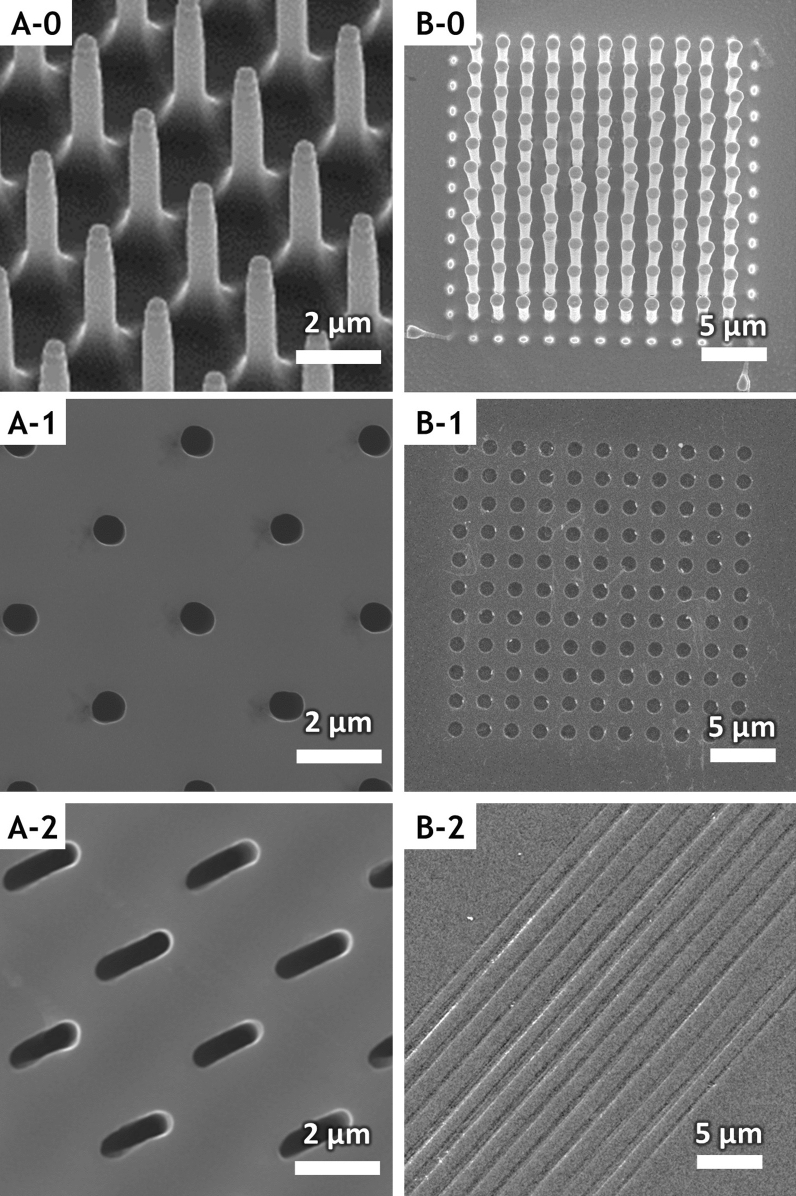
Metal catalyst probes and nano/microstructures on Si substrate after chemical imprinting and chemical carving. Two different probe arrays with a diameter of 600 nm (**A-0**) and 1 μm (**B-0**) were used. (**A-1**) Deep hole arrays with a depth of 1.5 μm were chemically imprinted on a Si substrate after etching for 30 min. (**A-2**) Individual trenches were fabricated via chemical carving by extending the holes in the direction of probe scanning. The scan direction does not match the alignment of the probe. (**B-1**) Shallow hole arrays with a depth of 80 nm were chemically imprinted on an Si substrate after etching for 10 s. (**B-2**) Individual trenches fabricated via chemical carving were combined in rows resulting in long trenches.

Figure 4 shows SEM images of the above-mentioned probes (A-0, B-0) and the corresponding patterns carved on the Si substrates, the circular hole array (A-1, B-1), and the trench array (A-2, B-2). Figure 4A-0 shows the pillar arrays with a diameter and height of 600 nm and 5 μm, respectively, with 2-μm spacing. Figure 4A-1 shows the hole arrays with a diameter and depth of 600 nm and 1.5 μm, respectively, clearly imprinted on the Si substrate after the probes were pressed on the substrate for 30 min in the etch bath. The substrate was pressed by the probe only vertically using the actuator, and the MaCE reaction on the substrate occurred in the downward direction. The diameters of the imprinted holes and probe pillars are approximately the same, suggesting that the vertical MaCE driven by the catalytic metal of the probe tip was the dominant mechanism. With the probe embedded in the Si, when probe scanning occurs in the horizontal direction, MaCE occurs at the side of the probe and the Si is laterally etched. As shown in Fig. 4A-2, the circular hole is elongated in the scan direction and a trench is eventually carved in the Si, suggesting that the catalytic metal attached to the side of the probe dominated the horizontal MaCE of the Si. Both ends of the trench retain a circular shape similar to the pillar of the probe used.

Figure 4B-0 shows the pillar array with a diameter and height of 1 μm and 5 μm, respectively, with a 1-μm spacing. Figure 4A-0,B-0 can be used to compare the results of chemical carving with pillar arrays of different diameters. Figure 4B-1 shows a circular hole array with a depth of 80 nm which was chemically imprinted after pressing the probe array in the vertical direction on the Si substrate for 10 s in an etch bath. The shallower imprinted depth is the result of a shorter chemical reaction time. Figure 4B-2 shows shallow trenches with a 1 μm width, 50 nm depth, and 300 μm length (all not shown in image) chemically carved in the Si substrate. The probe is horizontally scanned at a speed of 0.312 μm/s, and the appropriate force to maintain contact with the Si is applied vertically. The pillar probe scanning enabled continuous MaCE reactions with the Si, resulting in chemical carving of the shallow but long trench pattern shown.

**Figure 5:**
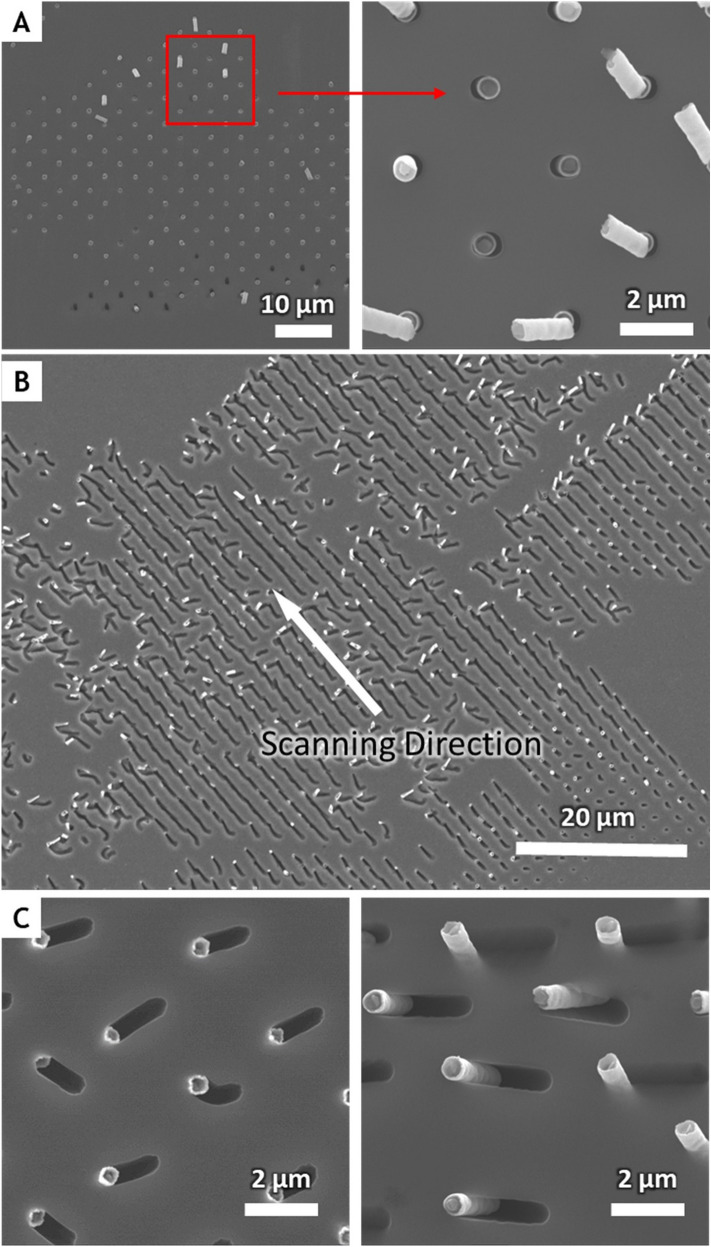
SEM images of metal catalyst probes plugged in Si substrate and the resulting holes and trenches. (**A**) Metal probes are plugged in the Si substrate. The probes were used to chemically imprint holes for 30 min after which they broke due to excessive stress. (**B**) The merged trenches indicate a slight zigzag pattern due to the inconsistency in the scan direction. Some probes are broken and remain in the Si substrate. (**C**) Horizontal carving occurs to some extent by the probe being scanned with twists, but it is also distorted in the downward direction due to excessive shear stress.

Figure 5 shows broken probes and the Si substrate etched using chemical carving. The probes were vertically etched and then deeply embedded in the substrate. These probes can be destroyed if they are scanned continuously with excessive horizontal force. The probe driven by the mechanical actuator moved at a rate faster than the etching rate of the chemical reaction, and a function for compensating for the deviation was not applied in the prototype. The incomplete chemical carving due to probe breakdown highlights the need for future studies in which heterogeneous materials can be selectively implanted in Si. In addition, the results obtained during the dynamic chemical carving can be used to further the microscopic understanding of the chemical carving mechanism.

Figure 5A shows an image captured after a significant vertical imprinting process at the moment when little horizontal carving occurred. Some probes are plugged into the Si after being broken, while others are tattered in the direction they are moving in. The cross section of broken probe shows the circular Si pillar, fluorocarbon protective layer, and Au catalyst.

Figure 5B shows the trenches formed and the broken probes after horizontal carving. As the scan progresses based on the spacing of the individual probes, the unit trenches formed from each hole are merged, forming a fairly long trench. A slight zigzag pattern can be observed in the trenches because the scanning direction and the alignment of the probes in the array do not match exactly.

Figure 5C shows trenches etched in different directions and angles. This indicates that the direction and angle of the scan are significantly different from the alignment of the probe. In this case, a larger torque may be applied to the probe to cause faster breakdown (Figs. S2, S3). Nonetheless, this suggests that the carving of a curved and oblique line is possible under the proper conditions by adjusting the scan direction and angle of the probe (Fig. S4). In addition, the gap between the trenches and pillars of 0.6 μm diameter plugged in the substrate is hard to notice. If we make metal catalyst probes small enough, we expect to fabricate tens of nm width structures with chemical carving. To minimize probe damage, vibration free table and precise mechanical controller are required. The fabrication of probes using materials that are harder and chemically more resistant than silicon, or flexible and intact, can also increase the durability of the probe. Once the probe damage issue is resolved, we expect that nm-scale probes show the benefits of faster etch rates and maximal scan rates because the products and reactants transport a shorter distance at the etch surface than sub-micrometer scale probes.

**Figure 6:**
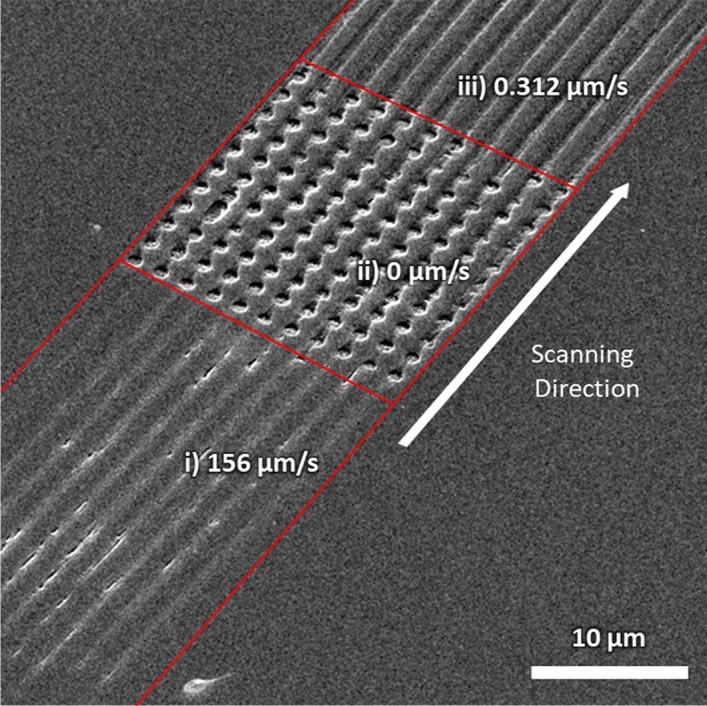
SEM image comparing the results of scanning with varying probe scanning speeds: (i) 156 μm/s, (ii) 0 μm/s for 10 s, and (iii) 0.312 μm/s. Non-uniform trench traces were formed in the region indicated by (i). Hole arrays are clearly imprinted in the region indicated by (ii) where the probe motion was paused. Some etched marks between the holes reflect the continuous scanning of the array before and after this region. Uniform trench arrays were chemically carved in the scanning direction in the region indicated by (iii).

Figure 6 shows the results of chemical carving using the pillar array for probing but with different scan rates. After aligning the scan direction of the probes of the array and the actuator, the scan was performed at three speeds. The actuator moved by 0.156 μm in one step and the scan rate was calculated based on the time before the next step. The array consists of 11 × 11 probes and the diameter of an individual probe is 1.0 μm.

Figure 6-i shows the image of a surface scanned at a scan speed of 156 μm/s (one step is movement by 0.156 μm followed by a pause for 1 μs). There is insufficient chemical etching, leaving only a trace of the probe being scanned. The individual probes were in continuous contact with the Si substrate, moving over a distance less than the diameter of the probes per step. However, there is insufficient MaCE at this scan rate.

Figure 6-ii shows the result of the actuator's motion being stopped for 10 s, that is, the probe and the Si were in contact in the etching solution for 10 s. Here, uniform holes were chemically etched by the probes. If it was a stationary probe, the results would be similar to those in like Fig. 4A-1,B-1, but owing to the series of fast-stop-slow moving probes, some etch marks remained between the holes.

Figure 6-iii shows the image of a surface scanned at a scan speed of 0.312 μm/s (one step is movement by 0.156 μm followed by a pause for 500 μs). The converted stationary contact time at the scanned region is calculated to 3.5 s using following relation. (Probe diameter/scan rate = 1 μm/312 nm/s ~ 3.5 s) The contact time with a specific region of the Si substrate was increased 500-fold compared to that in Fig. 6-i, which resulted in significant MaCE. Trenches with a uniform width and depth of 1 μm and 50 nm, respectively, were formed. Because of the continuous scanning of the array, the etch marks between the holes in Fig. 6-ii, become clearer with the movement in the scan direction. The scan speed of the probe controls the time for the chemical reaction, which determines the etch depth of the Si substrate. Interestingly, the etch rate was not linearly proportional to the etch time and depth of stationary contact region (8 nm/s at 80 nm depth pattern, 14 nm/s at 50 nm depth pattern) (Fig. S5). We believe that this is due to the increased diffusion distance and corresponding mass transport rate. Therefore, gray scale lithography through chemical carving is possible and requires slow scanning in the deep etched areas than in the shallow areas.

The engineering issue while studying chemical carving was a probe breakage. We believe that the durability of the probe can be improved in two ways by replacing the probe materials and introducing the feedback system to the actuator. First, instead of silicon used in this study, metal and polymer materials can be used to improve the strength and elasticity of the probes. Second, the contact-noncontact feedback system in the actuator can dynamically improve probe durability. Scanning faster than the etch rate causes probe breakage due to excessive pressure. Scanning after sensing the separation of the probe and substrate controls the scan rate and etching rate. This active feedback system can be established by sensing the interfacial potential shift between the metal catalyst and the etchant^29^.

## Conclusion

We presented a technology that can be used to produce nano-scale patterns on a Si substrate by scanning a catalyst metal probe in an etching solution. The new concept was demonstrated using prototypes. Pressure was applied to the probe in the vertical and horizontal directions, and the scan speed was controlled using an actuator. The Si substrate was etched via MaCE reactions using a probe in an etching solution. In the stationary mode, a circular pillar-shaped probe imprinted circular holes of the same shape in the Si substrate. In the scan mode, the probe carved trenches in the Si substrate by extending the holes in the scanning direction. The proposed chemical carving technique uses molds as in nano-imprint lithography; however, etching is performed directly without a polymer resist. In addition, this technology can be used for patterning using a computer numerical control based scanning technique, and simultaneous etching as low-cost alternative to conventional vacuum processing. However, without the precisely-controllable equipment, the probes are damaged and it is difficult to control the dimension of structure. It can be advanced further by developing more precise actuators and more durable probe materials.

## Methods

### Metal catalyst probe array preparation

A probe array was fabricated using boron-doped p-Si (100) substrates with a resistivity in the range of 5–30 Ω cm. The array pattern was defined on a spin-coated AZ5214 photoresist layer using contact aligner photolithography. A 1.4-μm-thick photoresist dot array pattern was used as an etching mask during dry etching using an inductively coupled plasma reactive-ion etcher (ICP-RIE). In order to establish steep probe profiles, a 2-step cyclic process of etching with sulfur hexafluoride gas (SF_6_) plasma and passivation with octafluorocyclobutane gas (C_4_F_8_) plasma (Bosch process) followed by an additional etching process using SF_6_ plasma to reduce the probe diameter. The polymer resist and fluorocarbon layer were cleaned via sonication in acetone, isopropyl alcohol (IPA), and deionized (DI) water for 5 min each. A 50-nm-thick fluorocarbon layer composed of Teflon-like material was deposited on the cleaned probe array as a chemical and electrical passivation layer using C_4_F_8_ gas plasma at 600 W for 30 s. A 100-nm-thick Au catalyst layer was sputtered on the fluorocarbon layer and annealed at 120 °C for 2 min on a hot plate (Supplementary Information 1).

### Chemical carving tool preparation

Motorized equipment was used for scanning at a uniform speed. The equipment includes a motorized stage that moves along the X- and Y-axes and a servo actuator that moves along the Z-axis and is operated by an Arduino board. The etch bath that contains the etching solution was made with Teflon and installed on the motorized stage such that it could be detached. The motorized stage was operated by a step motor and it moves 5 μm per step in the full step mode. The motor driver has a 32-resolution microstep feature and it was used to set the scan distance to 156 nm per step. Pulse signals were sent to the motor drive by the Arduino to operate the step motor. The speed was changed by adjusting the pulse intervals. A detachable probe holder was attached to the servo actuator, which can move upwards and downwards, and 40 N of force was applied. The motorized stage’s speed and the distance moved by the servo actuator in the upward and downward directions were adjusted by the Arduino using a customized code. The orders were entered using a keypad.

### Chemical carving

The semiconductor substrate was sonicated for 5 min in an acetone-IPA-DI water mixture to remove any organic matter. It was soaked in buffered oxide etchant for 1 min to remove the oxide film. Then, it was rinsed in DI water for 1 min and dried with N_2_ gas. The semiconductor substrate was fixed in the etch bath containing a mixed solution of H_2_O_2_:HF:DI water in a ratio of 1:10:10. The etch bath was installed on the motorized stage. The metal catalyst probe was attached to the probe holder using double-sided Kaptone tape and then attached to the servo actuator. The servo actuator was used to place the metal catalyst probe in the etch bath. The probe and the semiconductor substrate were in contact for ~ 10 s, after which the scanning began. The semiconductor substrate was separated from the stage after processing and rinsed for 5 min in DI water. After rinsing, the substrate was dried with N_2_ gas.

## Supplementary information

Supplementary file1
