# Supplementary material for: Chemical carving lithography with scanning catalytic probes
Source: Sci Rep. 2020 Aug 7;10:13411. doi: 10.1038/s41598-020-70407-1 (PMC7415144; doi:10.1038/s41598-020-70407-1)
Supplement: Supplementary file 1 — Supplementary file1 [file 41598_2020_70407_MOESM1_ESM.pdf]

Electronic Supplementary Information

Chemical Carving Lithography with Scanning Catalytic Probes

Bugeun Ki,[a,b] Kyunghwan Kim,[a,b] Keorock Choi,[a,b] and Jungwoo Oh[a,b]*
a. School of Integrated Technology, Yonsei University, Incheon 21983, Republic of Korea
b. Yonsei Institute of Convergence Technology, Incheon 21983, Republic of Korea

*Corresponding author: jungwoo.oh@yoensei.ac.kr

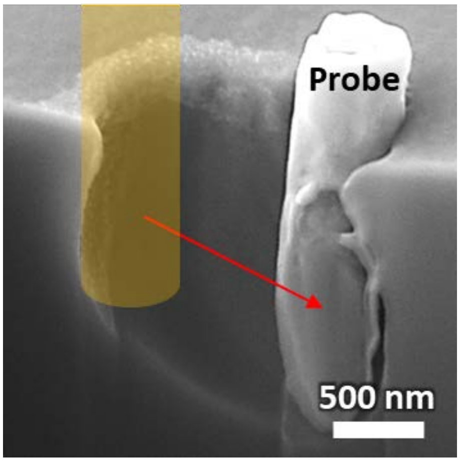

Figure S1. Cross-sectional SEM image of chemically carved Si and the embedded probe. The fluorocarbon layer in the probe is exposed upon ion-milling. Horizontal pressure is used to ensure continuous contact of the side of the probe and the Si substrate, and thus, a trench is chemically carved. The depth of the trench is increased by applying vertical pressure, which is difficult to control accurately in the prototype equipment.

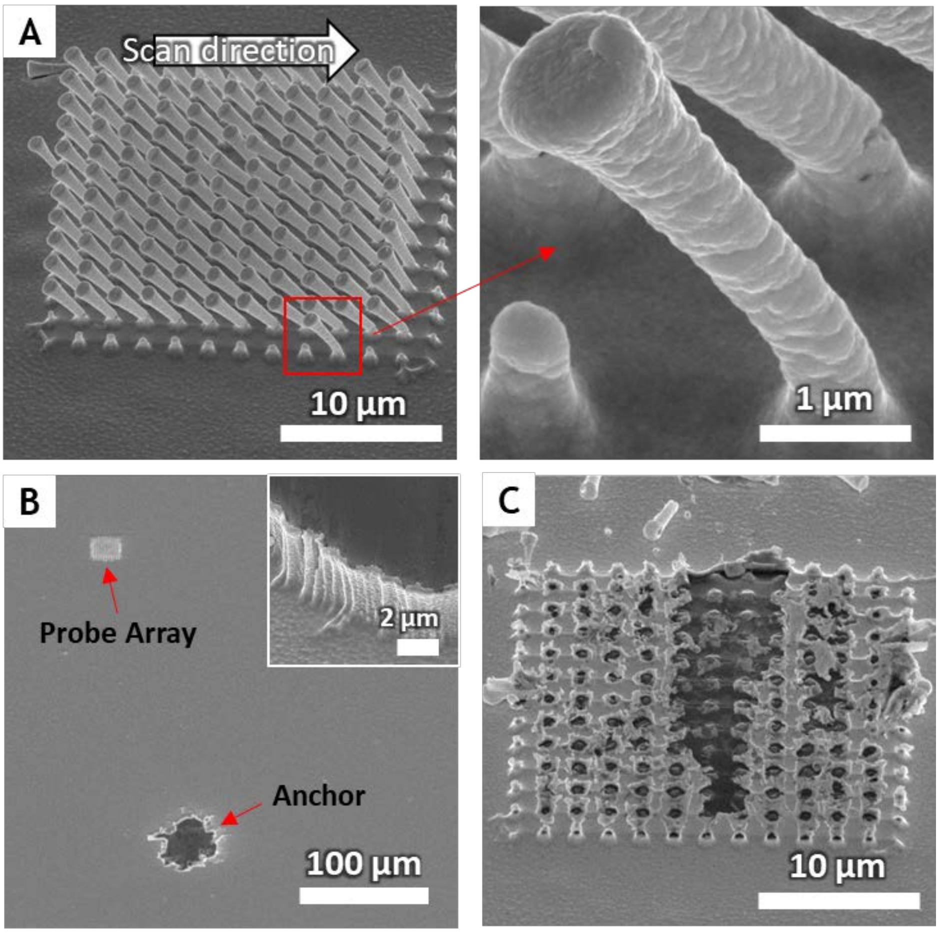

Figure S2. (A) SEM image of the probe array after chemical carving. The probes are bent in the direction opposite to the scanning direction due to the lateral pressure during scanning. The core of the metal catalyst probe, composed of crystalline Si, appears broken because of excessive bending of the probe. A ductile metal can be used to maintain the probe shape, because it will be flexible in the vertical and horizontal directions. (B) A structure of size 40 µm with the catalyst metal stripped (shown around the bent probe) was unintentionally formed by polymer residues during the etching process, but it prevented probe damage by dispersing the vertical pressure received by the probe. The fluorocarbon layer is exposed at the top of the structure and the metal layer appears peeled off during the chemical carving process. (C) Broken probes are absent. Due to the accuracy limitations of the z-axis servomotor of the prototype equipment, the probes were subjected to excess vertical pressure.

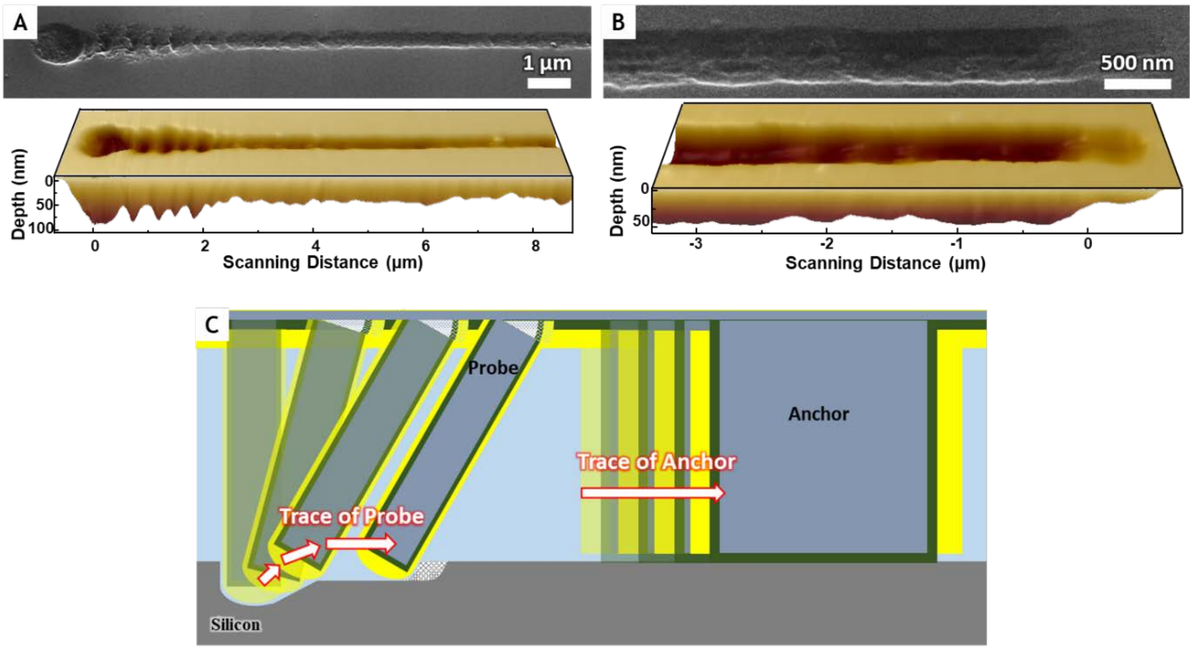

Figure S3. SEM and AFM images of the (A) start and (B) end of a 300-µm-long trench. The diameter of the hole at the beginning is 1 µm and the depth is 80 nm. (C) After the holes were formed, an irregular etching pattern was formed before a narrow and shallow uniform trench with a width of 500 nm and depth of 50 nm was carved during probe scanning. Such non-uniform etching occurs due to deformation resulting from the horizontal pressure during the initial scanning stage, when the probe is boring into the substrate. Although a high vertical pressure was applied due to the precision limit of the z-axis servo actuator, the anchor structure maintains the space between the Si substrate and the probe substrate, and the probe was warped but not broken. We should study probes with improved durabilities by applying the anchor structure in the future.

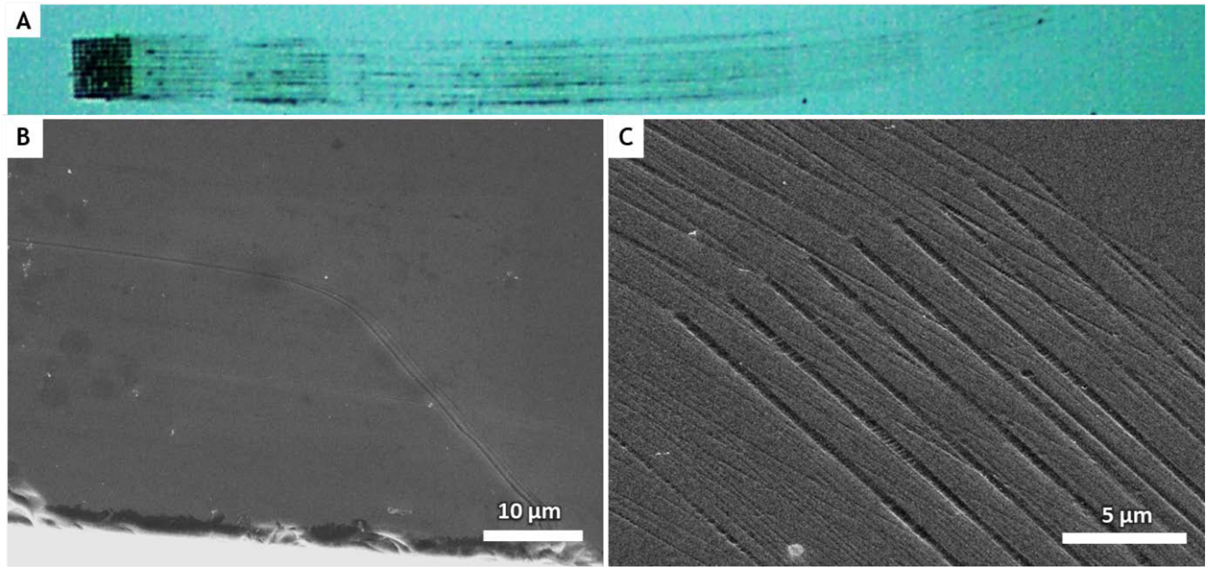

Figure S4. Carved trajectories are shown as the scan direction changes during chemical carving lithography. (A) Optical image showing a curved path formed by scanning after etching with a stationary probe array. The surface texture of the carved Si reduced the reflectance and became black Si. (B) Curved trench produced uniformly by scanning a single probe. (C) The carved depth changes with changing scan direction due to the mismatch between chemical reaction speed and movement speed.

Figure S4 shows the carving when the scan direction of the pillar array probes is switched by the actuator. An optical image (Figure S4 (A)) shows a curved path formed by scanning after etching with a stationary probe array. The surface texture reduces the reflectivity to black Si, which is reported by MaCE to produce a subwavelength structure for optical devices. Figure S4 (B) shows a uniform carving trajectory after scanning one probe, which apparently balances the mechanical resistance and the chemical reaction rate on the probe. Figure S4 (C) shows that as the probe array changes its scan direction, the carved depth also changes, probably due to the mismatch between chemical reaction speed and movement speed. If the distortion due to chemical etching and differences in scanning speed can be reduced, this technique can be used to carve in any direction depending on the scan direction of the probes.

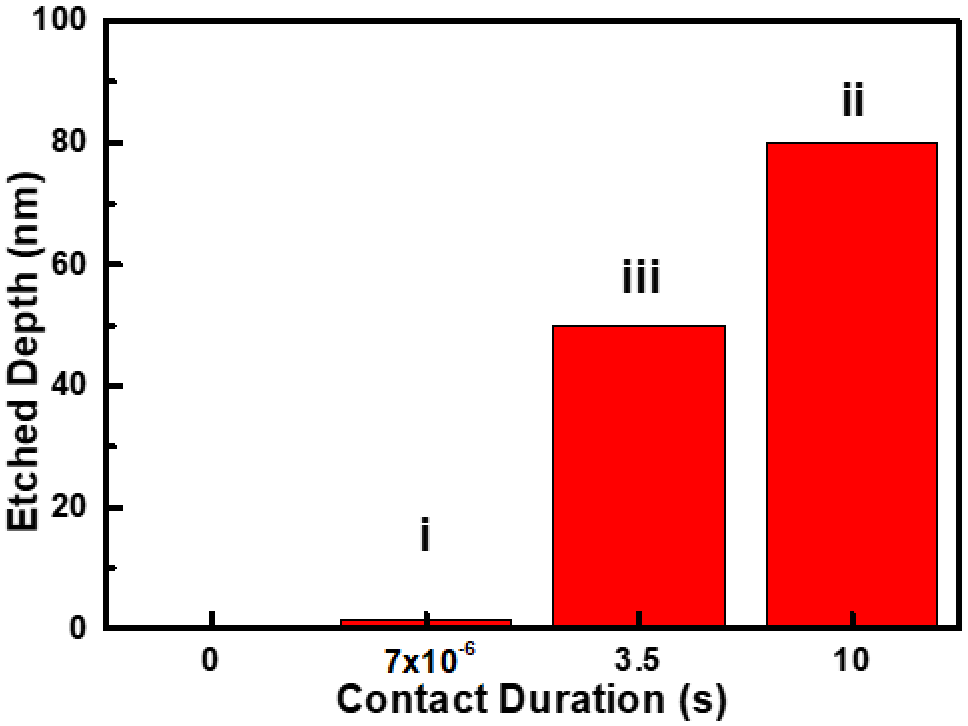

Figure S5. Etch depth in the areas of Figure 6 depending on different contact duration.

Figure S5 compares the etch depth as a function of contact duration as shown in Figure 6. The area without contact was not etched or showing corrosion. The contact duration in (i) region in Fig. 6 is estimated to 7 μs because the diameter of probe is 1 μm while the probe moves 0.156 μm for 1 μs. The surface in (i) region was hardly etched, showing the early stage of chemical carving. The estimated contact duration in (iii) region was 3.5 s and etch depth was 50 nm. The contact duration in (ii) region was three-fold of (iii) region but the etch depth was 80 nm.
